# Acupuncture Versus Biofeedback for Treatment of Functional Anorectal Pain

**DOI:** 10.5152/tjg.2024.22516

**Published:** 2024-02-01

**Authors:** Yahong Xue, Shuqing Ding, Huifen Zhou, Min Li, Jianbao Cao, Qian Chen, Yijiang Ding

**Affiliations:** 1 Nanjing Hospital of Chinese Medicine Affiliated to Nanjing University of Chinese Medicine, Nanjing, China; 2 Pelvic Floor Medicine Specialty Committee of World Federation of Chinese Medicine Societies, Nanjing, China

**Keywords:** Functional anorectal pain, acupuncture, biofeedback therapy, pelvic floor dyssynergia

## Abstract

**Background/Aims:**

Functional anorectal pain is one of several types of functional anorectal disorders. In this study, we compared the effectiveness of acupuncture (intervention) and biofeedback (control) as treatment for patients with functional anorectal pain.

**Materials and Methods:**

This prospective, single-center, randomized, and comparative study examined 68 patients with functional anorectal pain who were recruited from June 2017 to January 2019 at the Nanjing Hospital of Chinese Medicine Affiliated to Nanjing University of Chinese Medicine. Patients were randomly assigned to receive acupuncture or biofeedback. Patients in the acupuncture group received acupuncture at Zhongliao (BL33), Xialiao (BL34), Ganshu (BL18), Shenshu (BL23), and Dachangshu (BL25). Patients in the biofeedback group received pelvic floor biofeedback therapy, consisting of Kegel pelvic floor muscle training and electrical stimulation. Patients in both groups received 20 treatments over 4 weeks. The primary outcome was pain score on a visual analog scale, and the secondary outcomes were results from the MOS 36-item short-form health survey (SF-36) quality of life questionnaire, the self-rating depression scale, and the self-rating anxiety scale.

**Results:**

Visual analog scale pain scores significantly decreased in both of the groups with treatment (both *P* < .01). The final visual analog scale score was significantly lower in patients with pelvic floor dyssynergia who were treated with biofeedback (1.40 ± 0.97 vs. 5.30 ± 1.70) (*P *< .05). The 2 groups had similar decreases in self-rating depression scale and self-rating anxiety scale scores. Intriguingly, the acupuncture group had better mental health outcomes (*P* < .05).

**Conclusion:**

Both acupuncture and biofeedback therapy reduced the pain of patients with functional anorectal pain. Biofeedback provided more relief in patients with pelvic floor dyssynergia, and acupuncture provided greater improvements in mental health status.

**Main Points**

- This was the first study to compare the effect of acupuncture and biofeedback for functional anorectal pain (FAP), and both treatments were effective to relieve pain in patients with FAP.
- Biofeedback therapy had better efficacy than acupuncture in patients with pelvic floor dyssynergia.
- Acupuncture treatment relieved the pain of patients with FAP and also significantly improved the mental health of these patients.

## Introduction

Functional anorectal pain (FAP) is a non-organic disorder occurring in the anus and rectum, whose major symptoms are swelling, prickling, or burning pain.^1^ The prevalence rate of FAP in the general population is 7.7%.^1^ Multiple factors are involved in the pathogenesis of FAP, most of which are related to pelvic floor neuromuscular dysfunction. As a chronic and refractory anorectal disease, FAP has adverse effects on the health and quality of life of affected patients.^2,3^ Based on the duration of pain and the tenderness during traction on the puborectalis muscle, FAP can be subdivided into 3 categories: levator ani syndrome (LAS), unspecified functional anorectal pain (UFAP), and proctalgia fugax (PF).^4^ Current treatments for FAP are mainly non-surgical and include general therapies such as psychological counseling, sitting in a warm water bath, and application of drugs such as calcium antagonists, anti-spasmodics, analgesics, and antidepressants. However, the effectiveness of these therapies is uncertain because most publications of treatments for FAP were case reports or non-randomized controlled trials that did not provide robust evidence. 

Several recent studies examined the efficacy and advantages of acupuncture for the treatment of pelvic floor disorders. According to the theory of traditional Chinese medicine, acupuncture stimulates different meridians and modulates distal organs by “activating qi” and “promoting blood circulation,” thereby relieving pain. For example, 2 studies reported that needling at Baliao points significantly reduced the symptoms of defecation disorder, urinary incontinence, and pelvic pain.^5,6^ Although other studies demonstrated that acupuncture when used in combination with biofeedback therapy, herbal medicine, and other therapies, alleviated the symptoms of FAP, no randomized controlled study has yet examined the effect of acupuncture alone as a treatment for FAP.^7-9^ Thus the efficacy of acupuncture for FAP remains uncertain. Pelvic floor biofeedback therapy has been widely applied in patients with conditions such as outlet obstruction constipation, fecal incontinence, and LAS.^10^ A randomized controlled study showed that 87% of patients with LAS experienced symptom relief after biofeedback therapy.^11^ Thus, acupuncture and biofeedback both appear to have the potential for the treatment of FAP.

The present study compared the efficacy of acupuncture (intervention) with biofeedback (control) as a treatment for FAP. The general purpose was to optimize the therapeutic regimen and provide solid clinical evidence regarding the efficacy of acupuncture for FAP.

## Materials and Methods

### Design

**Figure 1.**
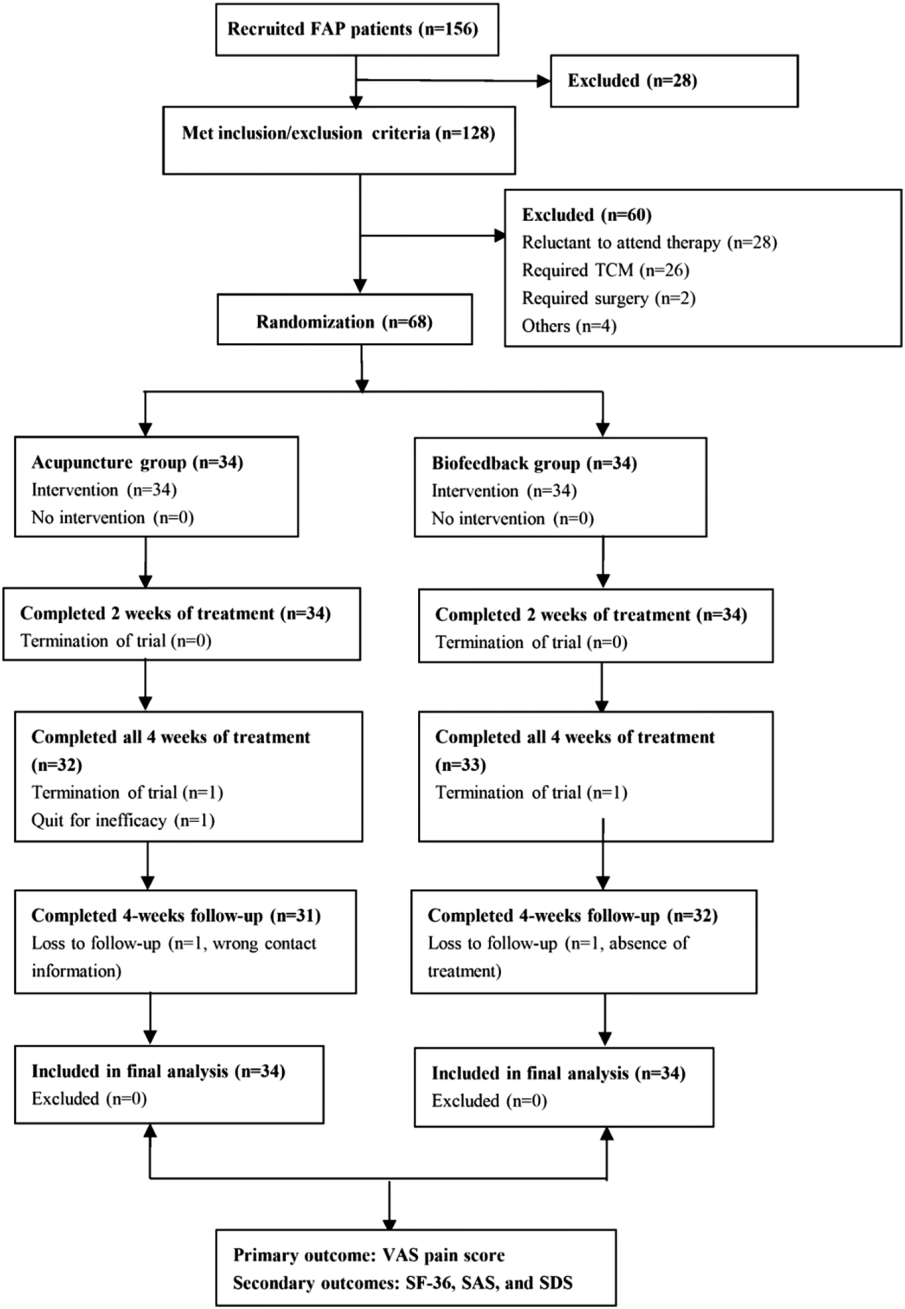
Flow chart of the study group.

This single-center, prospective, randomized comparative trial was designed according to the STRICTA criteria. The random number table and random number remainder grouping method were used to assign patients to an acupuncture group or a control group (pelvic floor biofeedback therapy). The trial consisted of 1 week of enrollment with baseline observations, 4 weeks of treatment, and 4 weeks of follow-up observations after treatment. Evaluation of treatment outcomes was assessed at the second and fourth weeks of treatment and at the end of follow-up (week 8, Figure 1). This trial was approved by the Ethics Committee of Nanjing Hospital of Chinese Medicine Affiliated to Nanjing University of Chinese Medicine (Approval No.: KY2016012) and all subjects signed informed consent agreements.

### Patients

Patients with FAP were enrolled in the Pelvic Floor Center of the Anorectal Department of the hospital from June 2017 to January 2019. Each included patient met the Rome IV diagnostic criteria for FAP,^12^ was 20-75 years old, signed the informed consent agreement, and was willing to cooperate during the trial. Patients were excluded if they had any of the following: pain caused by anal fissure, perianal abscess, hemorrhoids, or another organic anorectal disease; history of anal and pelvic floor surgery within the last 3 months; history of pelvic and spinal cord trauma; cardiovascular, cerebrovascular, liver, kidney, respiratory, or hematopoietic disease; a malignant tumor; presence of pregnancy, lactation, or menstruation; or diagnosis of any psychiatric disease or use of a psychiatric medication. Demographic data (gender, age, height, weight, and education level), disease course, pain characteristics (quality, location, and duration), concomitant symptoms, and physical examination results were recorded in detail.

### Interventions

Acupuncture was performed by 2 therapists, each of whom was licensed as a medical practitioner and had more than 5 years of work experience. Pelvic floor biofeedback therapy was performed by a therapist who had a biofeedback training certificate and more than 5 years of experience. Patients in each group received 5 treatments per week (30 minutes per treatment) during the course of 4 weeks.

In the acupuncture group, the main acupoints were Zhong Liao (BL33), Xia Liao (BL34), Ganshu (BL18), Shenshu (BL23), and Dachangshu (BL25). For Zhong Liao (BL33) and Xia Liao (BL34), both bilateral acupoints were selected and 75 mm needles were obliquely inserted into the acupoints (65 mm depth), so the tip formed an angle of 60° with the skin. Physicians were instructed to achieve de qi (an irradiating feeling deemed to indicate effective needling) if possible, and needles were stimulated manually at least once during each session. Electric acupuncture, with continuous waves at a frequency of 2/15Hz and the intensity of stimulation based on the patient’s comfort, was also applied to these 2 acupoints. For Ganshu (BL18), Shenshu (BL23), and Dachangshu (BL25), all bilateral acupoints were selected and 50 mm needles were perpendicularly inserted 40-50 mm into the acupoints. The acupuncture treatment used disposable sterile needles (0.32 mm × 50~75 mm; Huatuo, Suzhou Medical Supplies Factory Co., Ltd., Suzhou, China). The electric needle therapy used an SDZ.II instruments (Huatuo, Suzhou Medical Supplies Factory Co., Ltd.).

Patients in the pelvic floor biofeedback group received treatment with a pelvic floor surface electromyography biofeedback instrument (Thought-Technology, MyoTrac3, Canada Thought Technology, Canada). The patient was placed in an oblique supine position and was told to relax the whole body, separate the feet, and rotate the hips outward by 60°. Transanal electrodes (Nanjing Mailande Medical Technology Co., Ltd., model: MLD R2, Nanjing, China) were then applied. The 3 main training modes were relaxation training, Kegel pelvic floor muscle training (pelvic floor muscle contraction for 10 seconds, relaxation for 10 seconds), and electrical stimulation. The dominant mode was Kegel pelvic floor muscle training, whose aim was to restore the stability and coordination of the pelvic floor muscles. Treatment was performed for 30 minutes per day, 5 times per week, corresponding to 20 treatments over 4 weeks.

### Measurement of Outcomes

Pain, determined using a visual analog scale (VAS), was the primary outcome measure. In this scale, 0 indicated “no pain,” 1-3 indicated “mild pain,” 4-6 indicated “moderate pain,” and 7-10 indicated “severe pain.” Visual analog scale pain score was recorded at baseline, 2 weeks, 4 weeks, and 8 weeks.

The secondary outcomes were quality of life (QOL) and psychological well-being. Quality of life was scored using the simplified SF-36 life quality scale,^13^ in which a higher score indicates better status. The SF-36 was administered at baseline, 4 weeks, and 8 weeks and evaluates 8 dimensions: physical functioning (PF), role-physical (RP), bodily pain (BP), general health (GH), vitality (VT), social functioning (SF), role-emotional (RE), and mental health (MH). Psychological well-being was scored using the self-rating depression scale (SDS)^14^ and the self-rating anxiety scale (SAS),^15^ in which lower scores indicate better status. These 2 tests were administered at baseline and 4 weeks.

All evaluators were blinded to group allocations, received unified training, and were not allowed to ask patients for information other than that requested in the questionnaires.

### Statistical Analysis

The chi-square test was used to compare the demographic characteristics of the acupuncture and biofeedback groups. For the primary outcome measure (VAS pain score), the 2 groups were compared at 2 weeks, 4 weeks, and 8 weeks. The VAS pain score was also compared for patients who had LAS and UFAP, and 2 other concomitant pelvic floor diseases (pelvic floor dyssynergia and urinary incontinence). For the secondary outcomes, the scores on the SF-36 QOL assessment, SDS, and SAS were compared between groups and within each group at different times.

An intention-to-treat (ITT) approach was used for data analysis. The ITT dataset included all patients who were randomized. Patients who dropped out were followed up actively by telephone and text messages, and relevant data were recorded for statistical analysis. For missing data, records from the last follow-up before withdrawal were carried forward.

Statistical Package for Social Sciences (SPSS) 16.0 software (SPSS Inc.; Chicago, IL, USA) was used to establish the database and ensure data integrity and accuracy. Quantitative data were compared using a *t*-test or a non-parametric test. Counting data were compared using a chi-square test or Fisher’s exact probability test. A rank-sum test was used for the analysis of ranked data. All statistical tests were 2-sided, and a *P-*value below .05 was considered significant.

## Results

We initially recruited 156 patients who were admitted to the Pelvic Floor Center of the Anorectal Department of the Nanjing Hospital of Chinese Medicine Affiliated to Nanjing University of Chinese Medicine from June 2017 to January 2019 (Figure 1). Twenty-eight patients did not meet the inclusion/exclusion criteria, 28 patients were excluded because they refused any treatment, 26 patients were excluded because they were only willing to receive Chinese herbal medicine, 2 patients were excluded because they received surgery, and 4 patients refused participation for other reasons. The remaining 68 patients signed informed consent documents and were randomized to receive treatment. Sixty-three patients (92.7%) completed the trial, 3 patients (4.4%) terminated treatment midway, and 2 patients (2.9%) were lost to follow-up. All 68 patients were in the ITT dataset.

### Basic Characteristics of the Patients

Most patients were female (72.1%), and the average age was 56.91 years in the acupuncture group and 53.91 years in the biofeedback group (Table 1). Swelling pain was the major type of pain (73.5% in each group). Most patients had pain in the anus (70.6%) followed by pain in the perianal region (22.1%). Most patients had pain for 6-12 hours per day (41.2%) or 12-24 hours per day (35.3%), indicating that the enrolled patients suffered prolonged pain every day that significantly affected their daily lives. Many patients also had defecation disorders, fecal incontinence, urinary incontinence, or other pelvic floor dysfunctions. Levator ani syndrome (42.6%) and UFAP (50%) were the most common diagnoses in both groups, and PF (7.4%) was less common.

### Primary Outcome

Each group had a significant decline in VAS pain score from baseline to week 8 (both *P* < .01), but comparisons of the 2 groups indicated no significant differences at baseline, 2 weeks, 4 weeks, and 8 weeks (Table 2).

Stratification according to the type of pain (LAS vs. UFAP) indicated no significant difference in VAS pain scores between the 2 groups at baseline, 2 weeks, 4 weeks, and 8 weeks (all *P* > .05; Table 3).

We also analyzed the VAS pain scores of patients according to concomitant pelvic floor disease (pelvic floor dyssynergia vs. urinary incontinence; Table 4). Intriguingly, patients with pelvic floor dyssynergia who received biofeedback had significantly lower VAS pain scores at 4 weeks (*P* < .01) and 8 weeks (*P* < .05). However, patients with urinary incontinence in the 2 treatment groups had no significant difference in VAS pain score (*P* > .05).

## Secondary Outcomes

### SF-36 Quality of Life Score

Relative to baseline, the acupuncture group had significant improvements in the scores for PF, RP, RE, and MH at 4 weeks and 8 weeks and a significant improvement in the BP score at 8 weeks (all *P* < .05; Table 5). The biofeedback group had significant improvements in the scores for PF, RP, BP, GH, and RE at 4 weeks and 8 weeks (all *P* < .05). Acupuncture was superior to biofeedback therapy in improving MH at 4 weeks (*P* < .05) and 8 weeks (*P* < .01).

### Self-Rating Depression Scale and Self-Rating Anxiety Scale Scores

**Figure 2.**
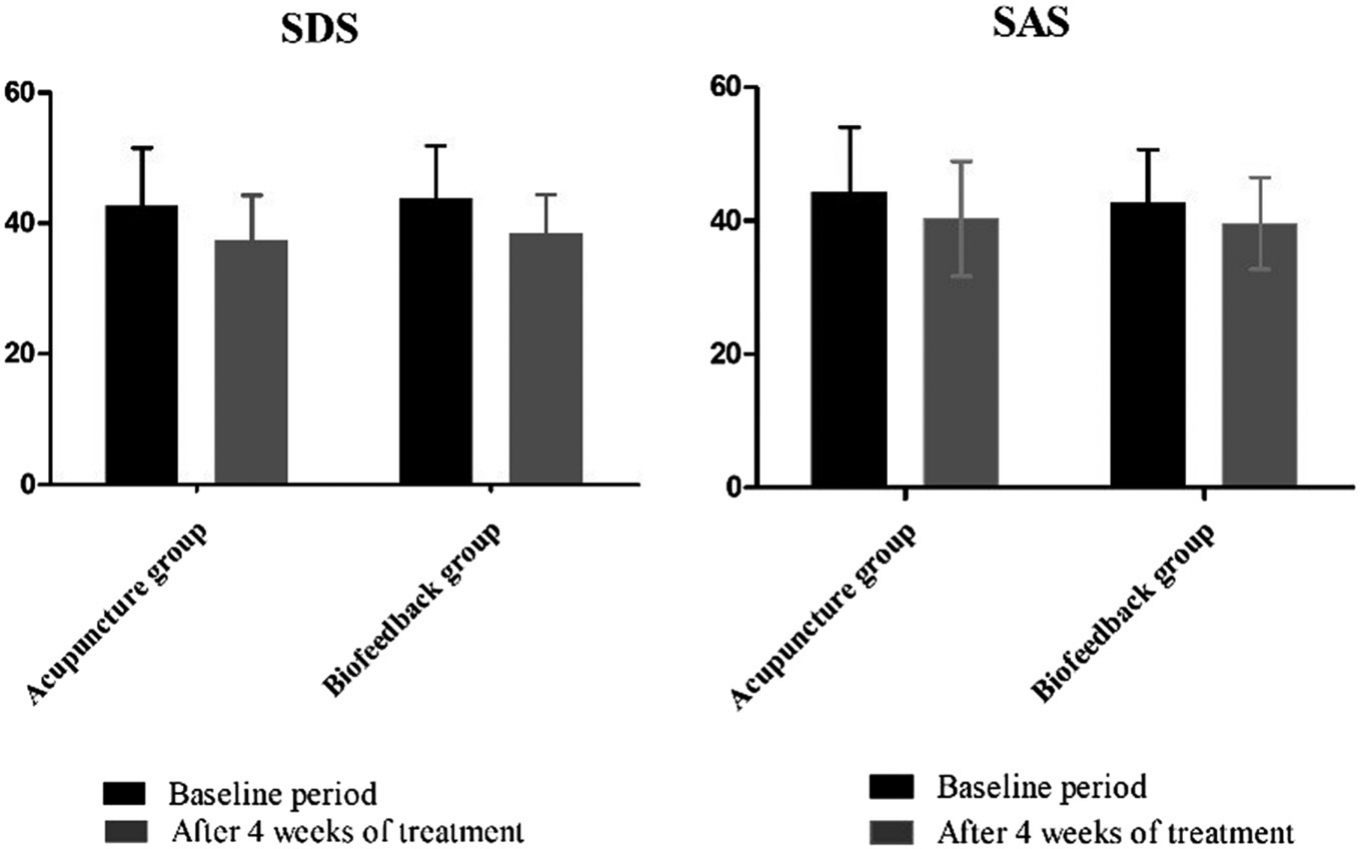
Self-rating depression scale and self-rating anxiety scale scores at baseline and 4 weeks in the 2 groups.

Compared with baseline, the SDS and SAS scores in both groups were significantly lower (indicating better status) at 4 weeks (all *P* < .01, Figure 2). However, there was no significant difference in SDS and SAS scores between the 2 groups at 4 weeks (all *P* > .05). 

## Discussion

Functional anorectal pain is a condition that is more common in women but whose pathophysiology is not well understood.^16^ In agreement, 72.1% of our patients with FAP were females. In addition, most of our patients experienced a long course of FAP, with an average duration of 40.94 months in the acupuncture group and 38.82 months in the biofeedback group. Previous research indicated that patients with a short course of the disease, especially those with proctalgia fugax, did not consider their condition seriously, in that only 17%-20% of these early stage patients consulted with doctors.^17^ Previous studies of FAP conducted by our department found that the incidences of LAS and UFAP were higher than that of PF.^9,18^ Atkin et al^19^ studied 170 patients with FAP and also showed that the prevalence of chronic anal pain (including LAS and UFAP) was 93%, and the prevalence of PF was only 7%. The majority of FAP patients experience swelling pain, described as “vague, dull pain or rectal compression” in a previous study.^20^ Functional anorectal pain is often accompanied by other pelvic floor disorders, including defecation disorders, urinary incontinence, pelvic organ prolapse, and fecal incontinence, and thus often requires multidisciplinary cooperation of health care providers for diagnosis and treatment. In addition, SDS and SAS evaluations of patients before treatment indicate that psychological factors play an important role in the pathogenesis of FAP. Thus, it is important for healthcare providers to consider the psychological problems in these patients.

Our results indicated that the VAS pain scores in both groups were significantly lower at 2 weeks, 4 weeks, and 8 weeks relative to baseline. Thus, acupuncture and biofeedback were both effective treatments that significantly relieved pain in these patients. In routine clinical practice, the acupuncture or biofeedback treatment program consists of 2 courses of treatment given over 4 weeks, a procedure considered to provide good efficacy. We found that acupuncture and biofeedback provided therapeutic effects after 4 weeks of treatment and after 8 weeks of follow-up. Our results indicated that biofeedback therapy had better efficacy than acupuncture in patients with pelvic floor dyssynergia, suggesting that this pain may be attributed to the uncoordinated movement of puborectalis muscle.^21^ Like biofeedback, acupuncture treatment relieved the pain of patients with FAP and also significantly improved the mental health of these patients. In agreement, a previous study showed that acupuncture at Ganshu (BL18) had analgesic effects due to its influence on the expression of brain-gut peptide substance P (SP), which affects mental states such as depression.^22^

The core acupoints in this study were Zhong Liao (BL33) and Xia Liao (BL34), which are part of the Baliao acupoints, which correspond to the posterior sacral foramen on each side. Targeting these acupoints may provide benefits because they presumably affect the first to fourth sacral nerves, which pass through the posterior sacral foramen and directly control pelvic floor muscles and intestinal functions. The acupoints at Shangliao (BL31), Zhong Liao (BL33), and Xia Liao (BL34) are most frequently used in clinical practice.^23^ Thus, deep puncture of Baliao could directly stimulate the sacral nerves, as in the modern therapy of sacral neuromodulation (SNM), which has been widely used to treat FAP and other pelvic floor disorders.^24-26^ Rongqing et al^3^ demonstrated that 75 of 120 patients with FAP (62.5%) who received SNM were cured, and the effectiveness was 96.7% at the 1-year follow-up. Roth^27^ applied SNM to treat anorectal pain with pelvic floor dyssynergia, and their results showed that the SNM significantly relieved pain and the symptoms of forced defecation and hand-assisted defecation. Biofeedback is the first-line option for the treatment of pelvic floor dysfunction,^28^ and Kegel pelvic floor muscle training is the most important and basic procedure. This procedure aims to improve the stability of pelvic floor muscles, reduce the excitability of pelvic floor sympathetic nerves, and relieve pelvic floor muscle spasms. Moderate electrical stimulation can activate the efferent fibers of the pudendal nerve, rebuild neuromuscular excitability, and enhance the contractility of pelvic floor muscles. Previous studies showed that acupuncture at the Baliao acupoint combined with biofeedback treatment had an effectiveness of 75% in the treatment of FAP.^9^

However, no previous studies performed head-to-head comparisons of acupuncture and biofeedback for the treatment of FAP. Most clinical trials of acupuncture performed comparisons with placebo controls. However, it is difficult to implement a placebo acupuncture treatment, so this approach is controversial.^29^ To truly assess the clinical efficacy of acupuncture, we used biofeedback therapy (an accepted treatment) as the control treatment, in accordance with the requirements of practical randomized controlled trials.^30^ Thus, the design of this study was closer to the conditions of routine clinical practice than previous studies, and this allowed us to provide an overall evaluation of acupuncture efficacy.

A limitation of this study was that it was a relatively small single-center comparative study. In spite of this limitation, this was the first study to compare the effect of acupuncture and biofeedback for FAP, and the results suggest the potential of another treatment option for patients with FAP. A larger multi-center randomized controlled study is needed to confirm the advantages of acupuncture and biofeedback therapy for these patients.

Our major result is that acupuncture and biofeedback effectively relieved pain in patients with FAP. Biofeedback had more advantages in treating patients with pelvic floor dyssynergia, and acupuncture appeared to be better for patients with obvious mental health problems. 

## Figures and Tables

**Table 1.** Characteristics of the Enrolled Patients

Characteristic	All Patients (n = 68)	Acupuncture (n = 34)	Biofeedback (n = 34)
Female	49 (72.1)	25 (73.5)	24 (70.6)
Age, years	55.41 ± 11.80	56.91 ± 11.14	53.91 ± 12.40
BMI	23.29 ± 2.87	22.99 ± 3.06	23.59 ± 2.68
Education level			
Senior high school and above	26 (38.2)	11 (32.4)	15 (44.1)
Junior high school and below	42 (61.8)	23 (67.6)	19 (55.9)
Duration of disease (months)	39.88 ± 51.39	40.94 ± 59.78	38.82 ± 42.27
Pain characteristics			
Swelling	50 (73.5)	25 (73.5)	25 (73.5)
Prickling	9 (13.2)	5 (14.7)	4 (11.8)
Burning	3 (4.4)	2 (5.9)	1 (2.9)
Colic	1 (1.5)	1 (2.9)	0
Throbbing	3 (4.4)	1 (2.9)	2 (5.9)
Spasm	2 (2.9)	0	2 (5.9)
Location of pain			
Inside the anus	48 (70.6)	23 (67.6)	25 (73.5)
Perianal	15 (22.1)	8 (23.5)	7 (20.6)
Rectum	2 (2.9)	1 (2.9)	1 (2.9)
Others	3 (4.4)	2 (5.9)	1 (2.9)
Duration of pain per day			
<3 hours	7 (10.3)	3 (8.8)	4 (11.8)
3-6 hours	9 (13.2)	5 (14.7)	4 (11.8)
6-12 hours	28 (41.2)	12 (35.3)	16 (47)
12-24 hours	24 (35.3)	14 (41.1)	10 (29.4)
Onset time			
Day	29 (42.6)	13 (38.2)	16 (47.1)
Night	8 (11.8)	2 (5.9)	6 (17.6)
Irregular	31 (45.6)	19 (55.9)	12 (35.3)
Concomitant disease			
Pelvic floor dyssynergia	19 (27.9)	9 (26.5)	10 (29.4)
Fecal incontinence	4 (5.9)	2 (5.9)	2 (5.9)
Urinary incontinence	21 (30.9)	11 (32.4)	10 (29.4)
Diagnosis			
LAS	29 (42.6)	13 (38.2)	16 (47.05)
UFAP	34 (50.0)	18 (53)	16 (47.05)
PF	5 (7.4)	3 (8.8)	2 (5.9)

Data are presented as n (%) or mean ± SD.

**Table 2.** VAS Pain Scores of the Groups during Treatment and Follow-up

Group	n	Baseline	2 weeks	4 weeks	8 weeks
Acupuncture	34	6.15 ± 2.49	3.94 ± 2.21^▲^	2.65 ± 1.95^▲^	2.53 ± 1.81^▲^
Biofeedback	34	6.00 ± 2.13	4.24 ± 2.12^▲^	2.65 ± 1.70^▲^	2.38 ± 1.72^▲^
*P*		.794	.582	1.000	.733

Data are presented as mean ± SD.

^▲^ *P* < .01: compared to baseline.

**Table 3.** VAS Pain Scores of the Patients with LAS and UFAP Receiving Acupuncture and Biofeedback Treatments

Type of Pain	Group	n	Baseline	2 weeks	4 weeks	8 weeks
LAS	Acupuncture	13	6.31 ± 1.97	4.46 ± 2.40	3.31 ± 2.01	3.15 ± 1.72
	Biofeedback	16	5.73 ± 1.79	3.53 ± 1.68	2.20 ± 1.26	1.93 ± 1.53
	*P*		.427	.242	.089	.058
UFAP	Acupuncture	18	5.89 ± 2.74	3.56 ± 2.06	2.33 ± 1.91	2.22 ± 1.93
	Biofeedback	16	6.06 ± 2.41	4.62 ± 2.42	2.75 ± 1.95	2.38 ± 1.59
	*P*		.847	.174	.534	.804

Data are presented as mean ± SD.

**Table 4.** VAS Pain Scores of the Patients with Pelvic Floor Dyssynergia and Urinary Incontinence Receiving Acupuncture and Biofeedback Treatments

Concomitant Disease	Group	n	Baseline	2 weeks	4 weeks	8 weeks
Pelvic floor dyssynergia	Acupuncture	8	6.75 ± 2.31	4.38 ± 2.26	3.38 ± 1.68	3.12 ± 2.10
	Biofeedback	10	5.30 ± 1.70	3.50 ± 1.43	1.40 ± 0.97	1.40 ± 1.17
	*P*		.145	.332	.006^▲^	.042^★^
Urinary incontinence	Acupuncture	11	6.12 ± 2.70	3.38 ± 2.33	2.12 ± 2.17	2.12 ± 1.81
	Biofeedback	10	5.75 ± 2.12	3.75 ± 1.91	2.38 ± 0.52	2.12 ± 0.84
	*P*		.762	.730	.756	1.000

Data are presented as mean ± SD.

Patients with pelvic floor dyssynergia receiving biofeedback compared with acupuncture, ^▲^ *P <* .01; ^★^ *P < *.05.

**Table 5.** SF-36 Indexes of the 2 Groups

Time	Group	PF	RP	BP	GH	RE	SF	VT	MH
Baseline	Acupuncture	71.57 (26.76)	39.71 (44.00)	52.58 (22.81)	41.03 (16.91)	41.18 (44.24)	65.69 (23.11)	64.26 (19.74)	67.29 (17.98)
	Biofeedback	75.33 (17.20)	31.62 (39.07)	49.74 (18.4)	35.29 (12.18)	38.24 (43.52)	58.50 (19.97)	62.21 (14.15)	63.76 (16.21)
	*P *(acupuncture vs. biofeedback)	.493	.426	.574	.113	.783	.175	.623	.398
4 weeks	Acupuncture	83.17 (23.94)^▲^	64.71 (39.46)^▲^	58.96 (23.49)	44.41 (20.40)	62.75 (42.45)^▲^	65.36 (23.97)	63.38 (23.08)	75.65 (15.46)^▲^
	Biofeedback	83.67 (17.96)^ ★^	55.15 (39.30)^★^	58.20 (21.75)^★^	44.12 (16.49) ^★^	65.69 (41.43)^★^	59.48 (25.93)	66.03 (20.74)	65.65 (19.07)
	*P *(acupuncture vs. biofeedback)	.922	.321	.891	.948	.773	.335	.621	.020^◆^
8 weeks	Acupuncture	77.61 (21.69)^▲^	69.85 (37.83)^▲^	63.46 (25.25)^▲^	47.65 (21.99)	68.62 (40.16)^▲^	68.30 (25.09)	65.59 (24.05)	77.65 (15.16)^▲^
	Biofeedback	84.67 (18.46)^★^	63.23 (42.74)^★^	59.64 (24.52) ^★^	45.29 (20.59)^★^	63.48 (44.66)^★^	63.40 (28.63)	67.21 (21.75)	63.41 (23.08)
	*P* (acupuncture vs. biofeedback)	.153	.501	.529	.650	.620	.455	.772	.004^◆^

Data are presented as mean (SD).

^◆^ *P* < .05: intergroup comparison.

^★^ *P* < .05: within-group comparison relative to baseline (biofeedback group).

^▲^ *P* < .05: within-group comparison relative to baseline (acupuncture group).

BP, bodily pain; GH, general health; MH, mental health; PF, physical functioning; RE, role-emotional; RP, role-physical; SF, social functioning; VT, vitality.
